# Electroencephalogram prediction of propofol effects on neuromodulation in disorders of consciousness

**DOI:** 10.3389/fneur.2025.1637647

**Published:** 2025-09-29

**Authors:** Xuewei Qin, Xuanling Chen, Xin Zhao, Lan Yao, Hongchuan Niu, Kai Li, Yuanli Zhao, Zhenhu Liang, Zhilei Lan, Yuqian Wang, Xiangyang Guo, Jiapeng Huang, Xiaoli Li

**Affiliations:** ^1^Department of Anesthesiology, Peking University International Hospital, Beijing, China; ^2^Department of Neurosurgery, Peking University International Hospital, Beijing, China; ^3^Institute of Electrical Engineering, Yanshan University, Qinhuangdao, China; ^4^Department of Anesthesiology, Peking University Third Hospital, Beijing, China; ^5^Department of Anesthesiology and Perioperative Medicine, University of Louisville, Louisville, KY, United States; ^6^State Key Laboratory of Cognitive Neuroscience and Learning & IDG/McGovern Institute for Brain Research, Beijing Normal University, Beijing, China

**Keywords:** electroencephalogram, disorders of consciousness, unresponsive wakefulness syndrome, propofol pharmacodynamics, Coma Recovery Scale-Revised, spinal cord stimulation

## Abstract

**Objective:**

This study aimed to characterize electroencephalogram (EEG) responses to low-dose propofol anesthesia in patients with disorders of consciousness (DoC) of distinct etiologies—traumatic brain injury (TBI), anoxic ischemic encephalopathy (AIE), and cerebrovascular accident (CVA)—and explore their prognostic relevance for recovery after spinal cord stimulation (SCS).

**Methods:**

A retrospective cohort of 40 DoC patients (TBI: 15, CVA: 14, AIE: 11) undergoing SCS under propofol anesthesia was analyzed. Pre- and post-anesthesia 19-lead EEG recordings were evaluated for power spectral density (PSD) in δ (0.5–4 Hz), θ (4–8 Hz), α (8–13 Hz), β (13–30 Hz), and γ (30–45 Hz) bands, alongside permutation entropy (PE). Consciousness levels were quantified using the Coma Recovery Scale-Revised (CRS-R) preoperatively and 3 months post-SCS. Etiology-stratified analyses compared neurophysiological and clinical outcomes.

**Results:**

Propofol universally suppressed β- (*p* < 0.001–0.05) and γ-band (*p* < 0.001–0.05) power across all groups. Etiology-specific EEG patterns emerged: AIE patients displayed reduced frontal α-power (Δ = −0.23, *p* = 0.03), while TBI/CVA patients showed prefrontal-parietal β/γ suppression (Δβ = −0.41, Δγ = −0.38; *p* < 0.001). Significant PE reduction (ΔPE = −0.21, *p* < 0.001) correlated with CRS-R improvement (*r* = −0.67, *p* = 0.003) in TBI/CVA subgroups but not in AIE (ΔPE = −0.05, *p* = 0.12). Three-month outcomes varied by etiology: 20% of TBI patients achieved a minimally conscious state (CRS-R ≥ 10) with enhanced motor (Δ = +0.25, *p* < 0.01) and visual function (Δ = +0.19, *p* = 0.03). CVA patients exhibited partial motor (Δ = +0.20, *p* = 0.007) and arousal gains (Δ = +0.17, *p* = 0.01), whereas AIE patients showed negligible improvement (mean ΔCRS-R = 0.4 ± 0.3).

**Conclusion:**

Propofol-induced EEG modulation reflects etiology-dependent neural network vulnerabilities in DoC. TBI/CVA patients demonstrated entropy reduction linked to clinical recovery, suggesting transient network stabilization that may enhance SCS efficacy. In contrast, AIE-associated static dynamics imply irreversible structural damage. Integrated PSD/PE analysis holds prognostic potential for predicting SCS responsiveness, particularly in TBI/CVA cohorts. These findings advocate etiology-tailored neuromodulation strategies, though multicenter validation is imperative for clinical translation.

## Introduction

Disorders of consciousness (DoC) encompass a spectrum of clinical syndromes ranging from coma to unresponsive wakefulness syndrome (UWS) and minimally conscious state (MCS) (1–4). These conditions typically result from severe brain injuries, including traumatic brain injury (TBI), stroke, and hypoxic encephalopathy (5, 6), and lead to impaired thalamocortical and cortico-cortical connectivity. Prolonged DoC refers to conditions where consciousness loss persists beyond 28 days, involving complex pathophysiological mechanisms that affect extensive brain networks (7, 8).

In clinical practice, the assessment and management of patients with DoC involve a complex multidisciplinary process that necessitates close collaborations among neurologists, rehabilitation physicians, anaesthesiologists, nurses, and the families of patients. Currently, effective methods to promote wakefulness in patients with UWS and MCS remain limited, as conventional pharmacological treatments show minimal efficacy (9–12). Moreover, current neurophysiological evaluations of DoC primarily depend on spectral analyses, such as power spectral density (PSD), which quantify oscillatory power. However, these methods demonstrate a limited ability to capture the nonlinear complexity of neural dynamics, a significant drawback considering the brain’s inherently nonstationary and adaptive characteristics (13, 14). For instance, while PSD can identify frequency-specific power changes, such as β/γ suppression, it cannot detect subtle modifications in signal regularity or network flexibility that may indicate residual functional connectivity in patients with DoC (15). To address this limitation, we proposed the integration of PSD with permutation entropy (PE), a model-free metric that quantifies temporal disorder, by examining ordinal patterns in EEG signals (16). This multimodal approach reconciles the analysis of oscillatory and non-oscillatory dynamics, providing a more comprehensive understanding of neurophysiological changes induced by anesthesia. Neuromodulation surgery has emerged as a promising treatment modality for DoC (17, 18), with SCS showing potential for enhancing the level of consciousness in patients with UWS (19–21).

SCS emerged as a therapeutic intervention for DoC based on its neuromodulatory effects on the ascending reticular activating system and thalamocortical circuits (19). Preclinical studies demonstrated that epidural cervical SCS increases cerebral blood flow and upregulates noradrenergic transmission in the locus coeruleus to facilitate cortical arousal (20, 21). Clinically, SCS combined with rehabilitation has shown the potential to improve consciousness levels in select patients with DoC, thus motivating its use in this patient cohort (19, 22).

As SCS implantation must be performed under general anesthesia, anaesthesiologists face significant challenges in determining the impact of general anesthetic drugs on the recovery of consciousness in such patients, as these agents have short- and long-term effects on arousal and cognitive function, potentially hindering recovery.

The mechanism of action of propofol, a widely used general anesthetic, is incompletely understood in the brain. Propofol primarily acts on GABA receptors, enhancing inhibitory GABAergic signaling and decreasing neuronal excitability (22, 23). On EEG, propofol induces dosage-linked slow δ-wave oscillations (24, 25). Rapid administration results in a shift from high-frequency, low-amplitude γ/β-waves characteristic of wakefulness to a high-amplitude, slow δ-wave pattern (26–28). This shift is associated with a reduced level of consciousness. Furthermore, the pharmacokinetic and pharmacodynamic characteristics of propofol must be considered to understand whether residual anesthetic affects patient EEG activity, complicating the monitoring and management of treatment strategies and efficacy in the clinical setting.

Additionally, the different aetiologies of DoC may cause varying brain responses to propofol. For focal brain injuries, propofol may primarily affect neural activity in regions adjacent to the injury, whereas, for diffuse brain injuries, its impact may extend to a broader range of brain regions. Propofol can affect the complexity and microstates of EEG activity, potentially reflecting the reorganization of the brain’s functional network (29).

These modifications in EEG activity are of clinical importance for evaluating patient consciousness level and functional brain state. Traditional frequency-domain analyses, such as PSD, can effectively capture changes in oscillatory power but may fail to account for the nonlinear dynamic properties of neural signals. To address this limitation, we have introduced PE, a nonlinear dynamical metric that quantifies the regularity of time series by analyzing ordinal patterns of adjacent data points (30). PE values, which range from 0 to 1, with higher values indicating greater randomness, reflect the brain’s capacity for adaptive information processing and network flexibility. By integrating PE with spectral analysis, this study aimed to provide a multidimensional characterization of propofol-induced neurodynamic changes in patients with DoC, thereby enhancing the understanding of etiology-specific neural resilience.

## Materials and methods

### General information

This retrospective cohort study was approved by the Ethics Committee of Peking University International Hospital (2024-KY-0062, Beijing, China) and was conducted in accordance with the Declaration of Helsinki. The study enrolled adult patients with DoC who received SCS electrode implants between January 2021 and April 2024 and whose representatives provided written informed consent.

The patients were categorized into three subgroups based on the pathophysiological mechanisms of their conditions: anoxic ischaemic encephalopathy (AIE), cerebrovascular accident (CVA), and TBI. As these differences may influence GABAergic receptor sensitivity to propofol, subgroup analysis was planned.

The inclusion criteria were (1) patients with DoC aged 18–60 years who met the International Classification of Diseases diagnostic criteria for DoC; (2) diagnosis of DoC, including UWS and MCS; (3) patients who required implantation of SCS electrodes during the study period; and (4) patients for whom consent was obtained from the legally authorized representatives for study participation.

The exclusion criteria were (1) severe cardiac, pulmonary, hepatic, renal, or other organ dysfunction; (2) coagulation dysfunction; (3) inability to obtain consent from the legal guardian; (4) known allergy to propofol or other medications related to the study; (5) history of psychiatric disorders before DoC onset; and (6) The concurrent administration of neuroexcitatory pharmacological agents, such as dopaminergic medications, zolpidem, or amantadine, or the engagement in alternative neuromodulation therapies, including transcranial direct current stimulation, repetitive transcranial magnetic stimulation, or vagus nerve stimulation, intended to enhance wakefulness or facilitate the recovery of consciousness within a three-month period preceding the implantation of SCS.

In this study, a senior neurologist confirmed the diagnosis through a comprehensive evaluation incorporating historical data, clinical presentation, and ancillary tests. The level of consciousness of patients with DoC was assessed preoperatively and 3 months postoperatively using the Coma Recovery Scale-Revised (CRS-R). The CRS-R comprises six subscales: auditory, visual, motor, orofacial/verbal, communication, and arousal. To ensure objective and consistent assessments, the scale was administered by professionally trained personnel. The scores were used to measure baseline consciousness levels and postoperative recovery outcomes.

### Anesthetic methods and perioperative management

The perioperative management of all patients in this study adhered to a standardized anesthetic protocol. Comprehensive preoperative monitoring was performed, including assessments involving electrocardiogram, pulse oximetry (SpO_2_), core temperature, and invasive radial artery blood pressure monitoring. After establishing invasive arterial access, blood samples were collected for blood gas and biochemical analysis.

For patients in a UWS, a respiratory circuit connected to a tracheal catheter was used to facilitate spontaneous ventilation. The anesthesia induction regimen included intravenous propofol (1 mg·kg^−1^) (31), sufentanil (0.3 μg·kg^−1^), and rocuronium bromide (0.5 mg·kg^−1^). Mechanical ventilation support commenced as soon as spontaneous respiration ceased. PetCO_2_ was monitored throughout the procedure and maintained within the target range of 35–45 mmHg.

During anesthesia maintenance, continuous infusion of propofol (1–1.5 mg·kg^−1^·h^−1^) and remifentanil (0.15 μg·kg^−1^·min^−1^) was administered via a micro pump. The propofol infusion rate was titrated to maintain a Bispectral Index (BIS) of 40–60 (BIS Vista™ Monitor, Medtronic, USA) (32). Hemodynamic parameters, including mean arterial pressure (MAP) and heart rate, were monitored in real-time via invasive arterial access. If the MAP decreased by >20% from baseline or the BIS was >60, the propofol infusion rate was increased by 0.2 mg·kg^−1^·h^−1^ every 5 min until the target parameters were achieved. Conversely, if BIS dropped to <40 or haemodynamic instability persisted despite vasopressor support, the infusion rate was reduced by 0.1 mg·kg^−1^·h^−1^. If the patient’s systolic blood pressure dropped by >20% from baseline during surgery, 3–6 mg of ephedrine was administered intravenously to raise the blood pressure. Norepinephrine infusion was initiated as needed. Based on arterial blood gas analysis, adjustments to the patient’s acid–base balance and electrolyte concentrations were made to keep them within normal physiological limits.

During surgery, patients with DoC were placed in the prone position. X-ray imaging was used to identify the T_7_–T_8_ gap, followed by needle puncture at 45° into the skin and epidural space. X-ray guidance facilitated electrode placement, ensuring that the upper edge of the electrode aligned with the midpoint of the C2 vertebral body in the epidural median. After confirming the normal impedance, the electrode was secured.

Following the procedure, propofol and remifentanil infusions were discontinued, and the patient was moved to the post-anesthetic recovery unit. Once spontaneous respiration returned, intravenous sugammadex sodium was administered to reverse rocuronium bromide. The patients were then transferred to the rehabilitation department for further treatment once SpO_2_ levels and circulatory stability met the pre-established criteria for discharge from the recovery room.

### EEG acquisition

**Figure 1:**
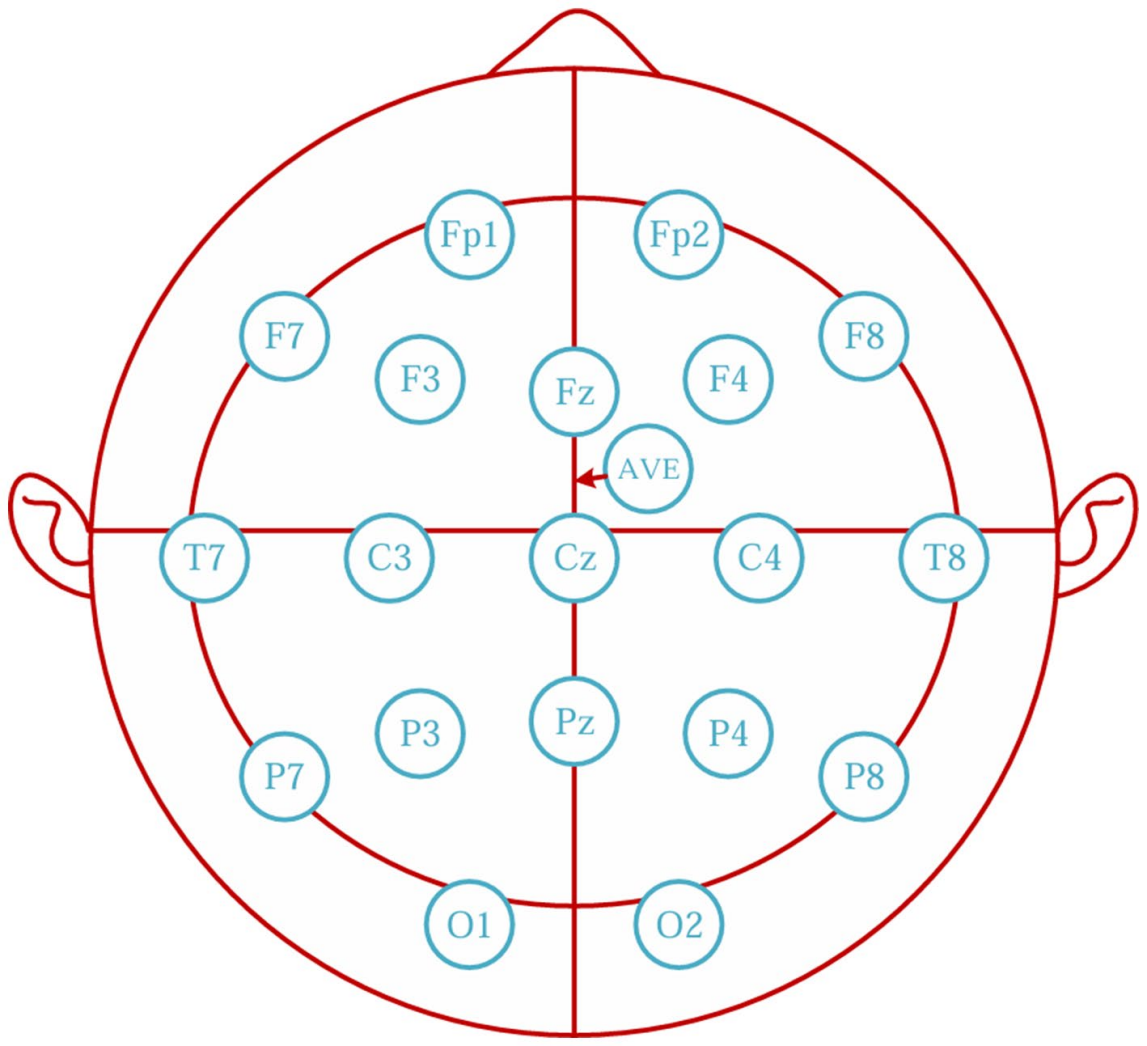
Distribution of 19-lead EEG electrodes. EEG, electroencephalogram.

To ensure EEG signal stability and reliability, an experienced neurologist positioned the disc electrodes in the patient’s ward 30 min before anesthesia. Disc electrodes were affixed to the patient’s scalp using a medical collodion to ensure secure adhesion. Medical mesh sleeves were used for additional fixation to minimize the risk of electrode dislodgement due to patient movement. A specialized conductive gel was also applied to maintain electrode impedance <5 kΩ to ensure signal stability and quality. EEG acquisition and monitoring were performed using a Nicolet system (Natus Medical Inc., USA), to guarantee accurate electrode placement, the neurologist meticulously followed the international 10–20 system guidelines, using anatomical landmarks and measurements to precisely position each electrode. The positioning process was carefully checked and double-checked by another expert to avoid errors. During prolonged monitoring, especially for patients with varying levels of consciousness, continuous monitoring of electrode status was conducted. Moreover, to address potential motion artifacts, several measures were taken. The medical mesh sleeves provided a tight but comfortable fit, allowing for some flexibility as the patient moved, thus reducing the risk of electrode displacement. The reference electrodes were averaged electrodes (AVE), and EEG signals were recorded using 19-lead electrodes (Figure 1). The specific lead positions included the left prefrontal (FP1), right prefrontal (FP2), left frontal (F3), right frontal (F4), frontal midline (Fz), left center (C3), right center (C4), left parietal (P3), right parietal (P4), and parietal midline (Pz). Additional electrodes were placed at the center midline (Cz), left occipital (O1), right occipital (O2), left anterior temporal (F7), right anterior temporal (F8), left mesial temporal (T3), right mesial temporal (T4), left posterior temporal (T5), and right posterior temporal (T6).

Scalp EEG recordings were acquired at four specified time points in relation to the administration of propofol: 1. Baseline (Pre-anesthesia): This was recorded 30 min after the placement of electrodes in the ward, prior to the administration of any anesthetic, with patients in a resting state. 2. Induction Phase: This phase commenced upon the completion of a propofol bolus (1 mg·kg^−1^) and continued until the cessation of spontaneous respiration, typically occurring 30–90 s post-injection. 3. Steady-State Anesthesia: During this phase, anesthesia was maintained throughout surgical stimulation (SCS electrode implantation), with the propofol infusion adjusted to achieve a target BIS of 40–60. A 5-min artifact-free segment was extracted after at least 15 min of stable BIS (±5 units). 4. Post-Recovery: This recording was conducted 30 min following the discontinuation of the propofol infusion in the post-anesthesia care unit (PACU), after the return of spontaneous respiration and the achievement of hemodynamic stability (mean arterial pressure within 20% of baseline).

The parameters for acquiring EEG signals were as follows: A linear phase finite impulse response (FIR) low-pass filter with a cut-off frequency of 0.5 Hz and an order of 1,000 was applied to exclude signals below the physiological EEG frequency range. Additionally, a high-frequency FIR filter with a cut-off frequency of 200 Hz and an order of 500 was used to eliminate potential high-frequency noise. A 2,000 Hz sampling rate was implemented to ensure high temporal resolution. Electrode impedance was continuously monitored in real-time using a dedicated impedance meter (Natus Medical Inc., USA). Adjustments were made using a conductive gel to maintain impedance levels <5 kΩ throughout the recording process.

### EEG data pre-acquisition and processing

The EEG data were pre-processed using the EEGLAB toolbox in MATLAB (version 2022a, MathWorks Inc., USA). The specific preprocessing steps were as follows:

**Elimination of baseline drift and head motion noise**: The pop_eegfiltnew.m function from the EEGLAB toolbox was used to apply a high-pass FIR filter, with a cut-off frequency of 0.1 Hz, utilizing a Hamming window and an order of 3,300, to mitigate low-frequency drifts. A zero-phase bidirectional filtering technique was implemented to prevent phase distortion.

**Elimination of high-frequency signal interference**: A linear phase FIR filter was applied to remove signal components with frequencies >45 Hz to mitigate high-frequency noise interference. While the filter reduced most high-frequency noise such as 50/60 Hz line noise, the remaining EMG artifacts (usually >50 Hz) were handled using independent component analysis (ICA) and visual inspection, as explained in the ‘Removal of apparent noise’section below.

**Downsampling**: The EEG signal was downsampled from 2,000 Hz to 100 Hz to decrease the computational load while preserving signal integrity, ensuring that the essential components of the EEG were maintained.

**Identification of poor-quality electrodes and problematic data segments**: Electrodes with poor conductivity and anomalous data segments were identified using visual inspection and automated detection algorithms. The veg plot.m function was used to visualize the data to allow clear identification of erroneous guides and problematic segments. These segments were removed through visual inspection, and interpolation was applied to repair any signal anomalies caused by electrode detachment or damage.

**Removal of apparent noise**: ICA was performed using the runica algorithm, based on the Infomax approach, within the EEGLAB environment. Artefactual components, such as those arising from ocular, muscular, or electrode noise, were identified by examining topographic maps, power spectra, and temporal characteristics. Artefactual components were classified using the following criteria:

1. Ocular artifacts, such as blinks and saccades, were identified by frontal scalp distribution (e.g., high weights in FP1/FP2 electrodes) and temporal waveforms correlated with eye movements. Validation was performed using the ADJUST toolbox (version 1.1.2) in EEGLAB, which detects ocular artifacts based on spatial and temporal features.
2. Muscular artifacts were characterized by broadband spectral power >30 Hz, particularly in the 50–150 Hz range, and transient high-amplitude spikes. These components were cross-validated against predefined EMG templates, focusing on temporal electrode dominance, using a correlation threshold of *r* > 0.7.
3. Electrode noise was identified using focal spatial topography, such as single-channel dominance or unstable impedance profiles detected through real-time monitoring.
4. Components meeting any of these criteria were manually excluded by two trained reviewers, with inter-rater agreement assessed using Cohen’s kappa (*κ* = 0.85) (33, 34).

**Re-referencing**: To enhance the signal quality and accuracy, the processed EEG data were re-referenced to an averaged reference.

Spectral analysis was performed using the multi-taper method, implemented in the Chronux toolbox (version 2.11; http://chronux.org/, accessed 8 September 2024). Time-frequency analysis was conducted across all EEG channels throughout the anesthetic period, focusing on the frequency range of 0.1–45 Hz. The brain regions were categorized into five areas: frontal (F3, Fz, F4), central (C3, Cz, C4), parietal (P3, Pz, P4), occipital (O1, O2), and temporal (T3, T4). The mean result of the time-frequency analysis for each region was calculated. Power spectra from the time-frequency analysis were compared across anesthesia states and divided into five frequency bands: δ-wave (1–4 Hz), theta-wave (4–7 Hz), α-wave (7–12 Hz), β-wave (12–30 Hz), and low gamma (30–45 Hz), with distinct background colors for differentiation. The multi-taper spectral parameters were as follows: window length = 5 s; overlap = 2.5 s, time-bandwidth product = three, and tapers = five.

### PE calculation

PE was calculated as follows:

(1) The time series {x(i):1≤i≤N} was reconstructed into an *m*-dimensional space: $X_{i}=[x(i),x(i+L),\ldots ,x(i+(m-1)L)]$ , where *m* is the embedding dimension and *L* is the time delay factor. The following equation was obtained by arranging X*_i_* in the increasing order:

(1) $$\left[x(i+(j_{1}-1)L)\leq x(i+j_{2}-1)L\leq \cdots \leq x(i+(j_{m}-1)L)\right]$$

In Equation 1, *x* is sorted by its corresponding *j* when identical elements exist.

(2) Because the embedding dimension is *m*, the sequence has a total of *m*! sorting modes. If the sorting mode is symbolized, a set of symbolic sequences can be obtained. The distribution probability of its sequences is calculated as $P_{1},P_{2},\ldots ,P_{k},k\leq m!$ . Based on Shannon’s entropy definition, the PE was calculated as follows:

(2) $$H_{p}(m)=\sum_{j=1}^{k}P_{j}\ln P_{j}$$

(3) The following is obtained after normalization:

(3) $$0\leq H_{p}=H_{p}/\ln(m!)\leq 1$$

where larger and smaller values indicate more complex and regular sequences, respectively. We choose *m* = 6 and *τ* = 1 as parameters to calculate the sequencing entropy (35–36).

### Evaluation criteria for consciousness improvement

The CRS-R scale was used to assess the potential for consciousness recovery in each patient with a DoC. Patients who met the specified criteria were considered to exhibit signs of consciousness improvement. Patients initially classified as UWS were deemed to have shown improvement if they attained MCS–, MCS+, or emerged from a minimally conscious state (EMCS). Similarly, patients initially classified as MCS– were considered to have shown improvement if they reached MCS+ or EMCS. If the clinical diagnosis after the treatment period did not indicate improvement compared with the initial assessment, the clinical outcome was classified as invalid (Table 1) (37, 38).

**Table 1:** CRS-R scale items.

Auditory		Visual		Motor		Oromotor/verbal function		Communication		Arousal	
Consistent movement to command	4	Object recognition	5	Functional object use	6	Intelligible verbalization	3	Functional: Accurate	2	Attention	3
Reproducible movement to command	3	Object localization: Reaching	4	Automatic motor response	5	Vocalization/oral movement	2	Non-functional: Intentional	1	Eye-opening w/o stimulation	2
Localization to sound	2	Pursuit eye movements	3	Object manipulation	4	Oral reflexive movement	1	None	0	Eye-opening with stimulation	1
Auditory startle	1	Fixation	2	Localization to noxious stimulation	3	None	0			None	0
None	0	Visual Startle	1	Flexion withdrawal	2						
		None	0	Abnormal posturing	1						
				None	0						

□, UWS; 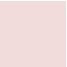, MCS−; 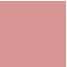, MCS+; 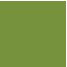, EMCS.

### Statistical analysis

All statistical analyses were conducted using IBM SPSS Statistics for Windows, version 22.0 (IBM Corp., USA) and R software (version 4.4.1).^1^ Before analysis, the normality of the data was assessed using the Kolmogorov–Smirnov test. For variables that did not meet the normality assumptions, such as CRS-R scores and PE values, non-parametric tests were applied. Between-group comparisons were executed using the Mann–Whitney U or Kruskal–Wallis tests, For statistical tests with multiple comparisons, the Benjamini–Hochberg procedure was used to apply false discovery rate correction to control for false-positive results, which is more flexible and reasonable than the Bonferroni correction and better preserves the power of statistical analysis, especially for exploratory research. Within-group changes, particularly those before and after anesthesia, were analyzed using the Wilcoxon signed-rank test. Categorical variables, such as diagnostic classification, were compared using Fisher’s exact test. To control for potential confounding variables, including age, sex, and baseline CRS-R scores, multivariate linear regression models were developed with delta permutation entropy (ΔPE) and CRS-R improvement as the dependent variables. The effect sizes were reported as Cohen’s d for parametric tests and rank-biserial correlation (r) for non-parametric tests. In the PE analysis, regional differences were assessed using mixed-effects models incorporating random intercepts for individual subjects. Two-way interaction analyses (group × anesthesia state) were performed, and *P*_FDR_ values were calculated. Pearson correlation was used to assess the link between ΔPE and ΔCRS-R scores, Linear regression quantified how well ΔPE explained ΔCRS-R, shown by the *R*^2^ value. The results are reported as mean ± standard deviation or median (interquartile range), and *p* < 0.05 was considered statistically significant.

## Results

### Patient characteristics and demographics

The study enrolled 40 adult patients with DoC who received SCS electrode implants during the study period. Among these 40 patients, the duration of neurological impairment before surgery ranged from 1 to 29 (median: 4) months.

The AIE, CVA, and TBI groups included 11, 14, and 15 patients, respectively. The Preoperative Coma Recovery Scale-Revised (CRS-R) total scores varied between 4 and 11 (AIE group: 4–7; CVA group: 5–11; TBI group: 6–10), with no significant differences in baseline scores across subgroups (*p* > 0.05).

The patients’ data are presented in Table 2. The preoperative general information did not differ significantly among the three groups (*p* > 0.05).

**Table 2:** Demographic features of the three patient groups.

Item	Sub item	Score	AIE group (*n* = 11)	CVA group (*n* = 14)	TBI group (*n* = 15)	*t*/*X*^2^	*p*
Age (year)			45.00 ± 11.46	44.67 ± 14.71	46.14 ± 10.23	0.055	0.947
Sex	M		9 (81.82%)	8 (57.14%)	11 (73.33%)	1.913	0.384
	F		2 (18.18%)	6 (42.86%)	4 (26.67%)		
Disease course (months)			3 (2.5.5)	5 (3, 6.5)	4 (2.25, 7.5)	0.867	0.648
Diagnosis	UWS		11 (100%)	11 (78.57%)	12 (80.00%)	2.689	0.261
	MCS–		0 (0%)	3 (21.43%)	3 (20.00%)		
	MCS+		0 (0%)	0 (0%)	0 (0%)		
CRS-R Total scores			6 (6,6.5)	7 (7,8)	7.5 (6,8)	5.816	0.055
CRS-R sub-item	Audio	0	3 (27.27%)	2 (14.29%)	3 (20.00%)		0.915
		1	8 (72.73%)	11 (78.57%)	12 (80.00%)		
		2	0 (0%)	0 (0%)	0 (0%)		
		3	0 (0%)	1 (7.14%)	0 (0%)		
		4	0 (0%)	0 (0%)	0 (0%)		
	Visual	0	7 (63.64%)	5 (35.71%)	1 (6.67%)	11.426	0.076
		1	3 (27.27%)	4 (28.57%)	5 (33.33%)		
		2	1 (9.09%)	4 (28.57%)	6 (40.00%)		
		3	0 (0%)	1 (7.14%)	3 (20.00%)		
		4	0 (0%)	0 (0%)	0 (0%)		
		5	0 (0%)	0 (0%)	0 (0%)		
	Motor	0	0 (0%)	0 (0%)	0 (0%)		
		1	4 (36.36%)	4 (28.57%)	2 (13.33%)		0.660
		2	7 (63.64%)	9 (64.29%)	12 (80.00%)		
		3	0 (0%)	1 (7.14%)	1 (6.67%)		
		4	0 (0%)	0 (0%)	0 (0%)		
		5	0 (0%)	0 (0%)	0 (0%)		
		6	0 (0%)	0 (0%)	0 (0%)		
	Oromotor/verbal function	0	1 (9.09%)	1 (7.14%)	0 (0%)		0.242
		1	10 (90.91%)	11 (78.57%)	15 (100%)		
		2	0 (0%)	2 (14.29%)	0 (0%)		
		3	0 (0%)	0 (0%)	0 (0%)		
	Communication	0	11 (100%)	14 (100%)	15 (100%)		1.000
		1	0 (0%)	0 (0%)	0 (0%)		
		2	0 (0%)	0 (0%)	0 (0%)		
	Arousal	0	2 (18.18%)	0 (0%)	0 (0%)		0.198
		1	0 (0%)	1 (7.14%)	1 (6.67%)		
		2	9 (81.82%)	13 (92.86%)	14 (93.33%)		
		3	0 (0%)	0 (0%)	0 (0%)		

UWS, unresponsive wakefulness syndrome; MCS, minimally conscious state; CRS-R, Coma Recovery Scale-Revised; m, month; TBI, traumatic brain injury; CVA, cerebrovascular accident; AIE, anoxic ischemic encephalopathy.

### Changes in prefrontal and frontal EEG temporal frequency spectra before and after anesthesia

**Figure 2:**
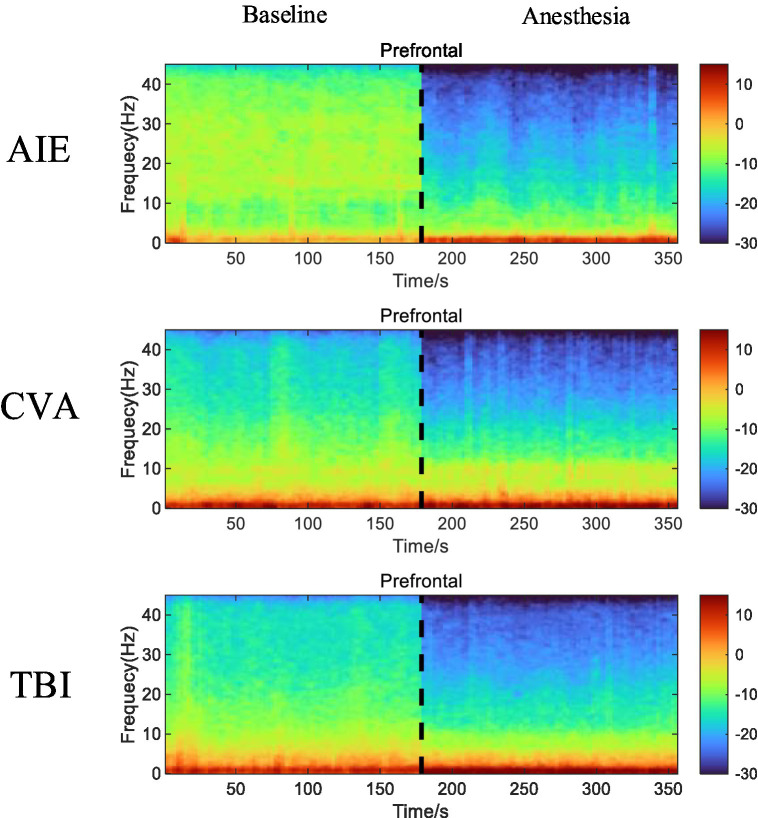
Time-frequency alterations in EEG activity in the prefrontal and frontal regions of the three patient groups before and after anesthesia with low-dose propofol. Compared with EEG activity recorded before anesthesia, the AIE and TBI groups display a marked reduction in β- and γ-wave (high-frequency bands) power and increased δ- and θ-wave (low-frequency bands) power in both the prefrontal and frontal regions. The CVA group also demonstrates a significant decrease in β- and γ-wave power in the prefrontal region and a decrease in γ-wave power in the frontal region, with minimal changes in the δ- and θ-waves. The CVA group exhibits EEG activity with features similar to those of *α*-waves in both the prefrontal and frontal regions. In the time-frequency spectrum, increased energy is represented in red, while decreased energy is represented in blue. EEG, electroencephalogram; AIE, anoxic ischemic encephalopathy; TBI, traumatic brain injury; CVA, cerebrovascular accident.

After low-dose propofol administration, all patient groups exhibited reduced β and γ EEG power in the prefrontal and frontal regions (*p* < 0.05). The AIE and TBI groups also showed increased δ-wave and θ-wave power (*p* < 0.05), while the CVA group experienced a decrease in β- and γ-wave power in the prefrontal region and only a γ-wave decrease in the frontal region, with no significant δ- and θ-wave changes. The CVA group maintained similar α-wave activity in both regions (*p* < 0.05). The PSD of EEG activity in the prefrontal and frontal regions of the AIE and TBI groups decreased in the high-frequency bands (β and γ) and increased in the low-frequency bands (δ and θ), particularly in the TBI group. The reduction in γ-wave power in the frontal region was especially pronounced, whereas changes in δ and θ were less marked (*p* > 0.05). In the CVA group, the PSDs of the β and γ were significantly reduced in the prefrontal region. The CVA group also showed EEG activity in both the prefrontal and frontal regions that resembled α-wave characteristics (Figure 2).

### Changes in EEG topography before and after anesthesia

**Figure 3:**
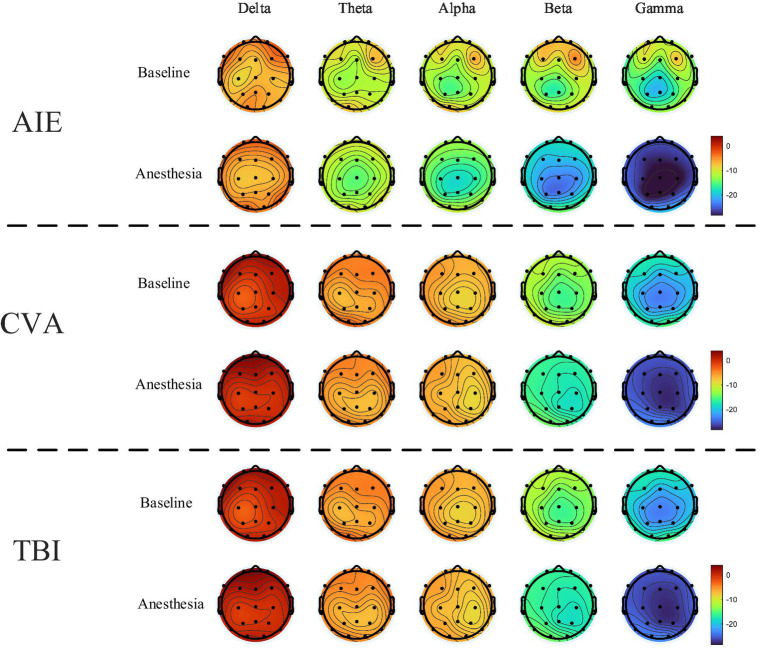
EEG topography of the entire brain before and after anesthesia at varying frequencies in the three patient groups. The power spectra of β- and γ-waves show marked declines in all groups following anesthesia compared with the pre-anesthesia recordings. Conversely, the θ-, δ-, and α-waves exhibit minimal variation before and after anesthesia. The color scale in the topography represents the relative power of each frequency band, with warm and cool colors denoting increased and decreased power, respectively.

After low-dose propofol administration, significant β and γ power suppression occurred in prefrontal (FP1/FP2) and frontal (F3/F4/Fz) regions across all groups (all PFDR < 0.05; Figure 2). AIE group: Increased δ (*p* = 0.02) and θ power (*p* = 0.03); TBI group: Increased δ (*p* = 0.01) and θ power (*p* = 0.007); CVA group: Isolated γ suppression in frontal regions (*p* < 0.001) without δ/θ changes (Pδ = 0.21; Pθ = 0.41) (Figure 3).

### Segmental spectra

**Figure 4:**
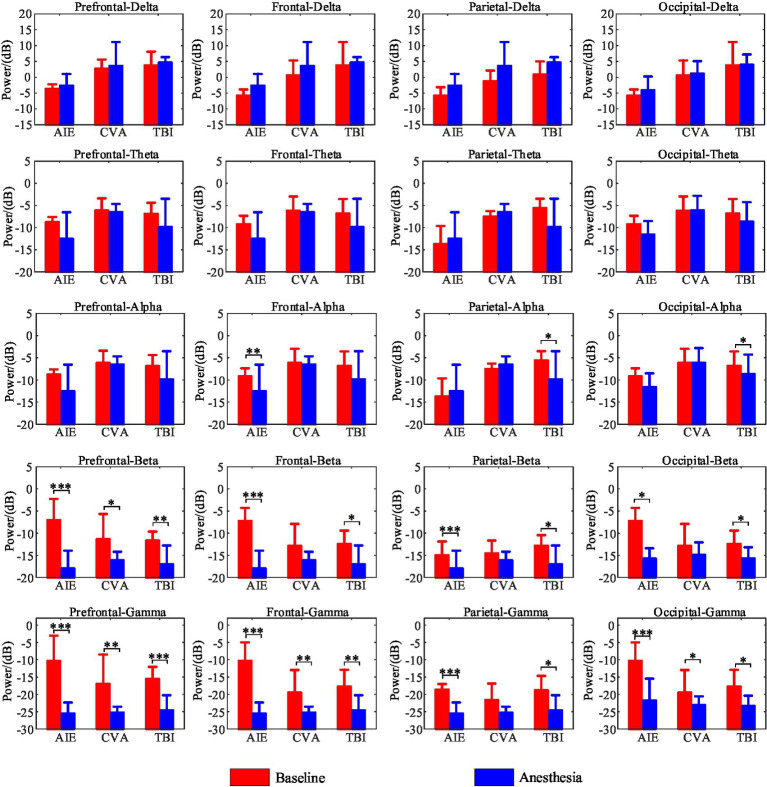
Changes in EEG wave power across brain regions pre- and post-anesthesia. In this study, we examined the alterations in power (measured in decibels) across five frequency bands in various brain regions of patients with three distinct types of disorders of consciousness, following administration of low-dose propofol, to elucidate its differential neuromodulatory effects. All etiological groups exhibited significant attenuation in beta and gamma power (*p* < 0.05, *p < 0.01, **p < 0.001), particularly within the anterior and frontal lobes, with the gamma band experiencing a maximum reduction of −25 dB. Specifically, the TBI group demonstrated the most pronounced suppression of beta and gamma power across the entire brain (prefrontal gamma: **p < 0.001), along with a decrease in parietal and occipital alpha power (*p* < 0.05). The CVA group showed selective suppression of prefrontal beta and gamma power (**p < 0.01) while preserving parietal gamma power (*p* > 0.05). The AIE group exhibited abnormally elevated prefrontal delta power (+15 dB, **p < 0.001) and a reduction in frontal delta power (−25 dB, **p < 0.001), with frontal alpha power also significantly attenuated (−18 dB, **p < 0.001). Furthermore, the TBI group experienced an enhancement in whole-brain delta and theta power (prefrontal delta: **p < 0.001), whereas the CVA group showed no significant changes in delta and theta power (*p* > 0.05), indicating a specific sensitivity of traumatic brain injury to slow wave activity. *p < 0.05, **p < 0.01, ***p < 0.001. TBI, traumatic brain injury; CVA, cerebrovascular accident; AIE, anoxic ischemic encephalopathy.

At the whole-brain level, θ- and δ-wave power did not differ significantly before and after anesthesia among the three patient groups (*p* > 0.05). However, the AIE group showed significantly decreased, α-wave power in the frontal region following anesthesia (*P*_FDR_ = 0.03). Similarly, the TBI group showed reduced α-wave power in the parietal and occipital regions (*p* < 0.05), but no other brain regions (*p* > 0.05). The AIE and TBI groups demonstrated significantly decreased β-wave power across the entire brain (*p* < 0.001–0.05), whereas the CVA group showed a significant decrease only in the prefrontal region (*p* < 0.05). Except for the parietal region in the CVA group, all brain regions in the three groups showed significantly reduced γ-wave power following anesthesia (*p* < 0.001–0.05) (Figure 4).

### Entropy measurements across brain regions

**Figure 5:**
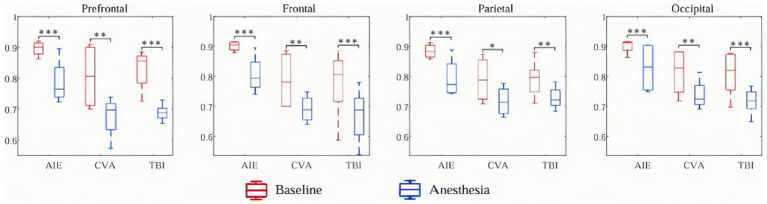
Regional PE changes pre-UWS post-propofol administration. The AIE group exhibits higher baseline entropy (PE = 0.72 ± 0.08) with a minimal reduction in entropy (ΔPE = −0.05, *p* = 0.12); where ΔPE denotes the PE change between pre- and post-anesthesia, calculated as ΔPE = PE(Anesthesia) − PE(Baseline). Conversely, the TBI/CVA group shows significantly decreased prefrontal entropy (ΔPE = −0.21, *p* < 0.001), which correlated with CRS-R score improvement (*r* = −0.67, *p* = 0.003). PE, permutation entropy; AIE, anoxic ischemic encephalopathy; TBI, traumatic brain injury; CVA, cerebrovascular accident.

In the anaesthetized state, all brain regions of patients across the three etiological subgroups exhibited decreased entropy relative to the preoperative baseline state. Notably, the AIE group demonstrated higher entropy levels than the other two groups under identical conditions. The decreased entropy was particularly pronounced in the prefrontal and frontal brain regions following anesthesia compared with the parietal and occipital regions (Figure 5).

### Changes in the state of consciousness of patients with DoC 3 months postoperatively

Table 3 shows the results of the comparisons of preoperative and 3-month postoperative level of consciousness among the three patient groups.

**Table 3:** Preoperative and 3-month postoperative levels of consciousness according to patient group.

Item	AIE group (*n* = 11)	CVA group (*n* = 14)	TBI group (*n* = 15)	Statistic	*p*
Diagnosis _(pre-operation)_				2.689	0.261
MCS–	0 (0%)	3 (21.43%)	3 (20%)		
UWS	11 (100%)	11 (78.57%)	12 (80%)		
Diagnosis _(3months)_				16.865	0.010
EMCS	0 (0%)	1 (7.14%)	3 (20%)		
MCS+	0 (0%)	3 (21.43%)	4 (26.67%)		
MCS–	0 (0%)	5 (35.71%)	4 (26.67%)		
UWS	11 (100%)	5 (35.71%)	4 (26.67%)		

UWS, unresponsive wakefulness syndrome; MCS, minimally conscious state; EMCS, emerged from a minimally conscious state; CRS-R, Coma Recovery Scale-Revised; TBI, traumatic brain injury; CVA, cerebrovascular accident; AIE, anoxic ischemic encephalopathy.

### Functional recovery of CRS-R sub-items 3 months postoperatively

**Figure 6:**
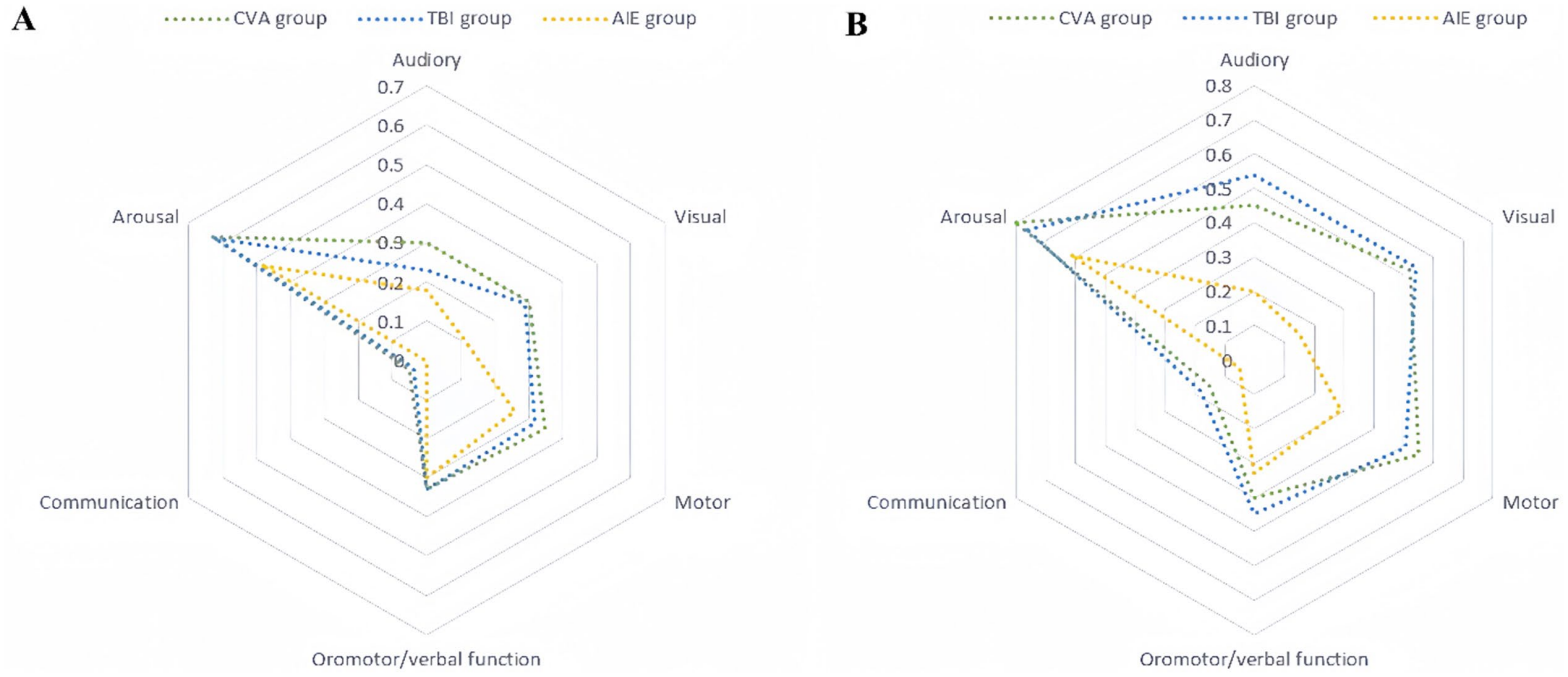
Standardized CRS-R subscores preoperatively and 3 months postoperatively. The standardized scores are presented on a 0–1 scale, where 0 denotes the lowest level of functioning and 1 denotes the highest. The radar charts comprise six axes, each representing one of the CRS-R subscales (auditory, visual, motor, oral/verbal, communication, and arousal). The units along these axes are standardized scores, which are dimensionless and derived by linearly transforming raw scores into the 0–1 range. The specific mapping relationships for each subscore are as follows: auditory (0–4 mapped to 0–1), visual (0–5 mapped to 0–1), motor (0–6 mapped to 0–1), oromotor/verbal function (0–3 mapped to 0–1), communication (0–2 mapped to 0–1), and arousal (0–3 mapped to 0–1). **(A)** Preoperative standardized CRS-R subscores. The plot shows three distinct groups: cerebrovascular accident (CVA, blue), traumatic brain injury (TBI, green), and hypoxic–ischemic encephalopathy (AIE, red). **(B)** Standardized CRS-R subscores at 3 months postoperatively. The definitions and standardization methods are the same as those in **(A)**. CRS-R, Coma Recovery Scale-Revised.

The radar charts of standardized CRS-R sub-scores (Figures 6A,B) showed significant differences in the recovery of consciousness before and 3 months after surgery.

Postoperative assessment in the TBI group revealed significant enhancements across multiple dimensions, with marked improvements in motor (from 0.32 to 0.51), visual (from 0.29 to 0.54), and auditory (from 0.23 to 0.54) functions, with change ranges (Δs) > 0.19 and statistical significance (*p* < 0.05). These findings suggest robust neuroplasticity. Notably, some patients achieved full consciousness, with two individuals attaining EMCS. Their motor function scores reached the maximum value of 1.0, and the postoperative radar chart approximated a regular hexagon, indicative of a well-rounded multidimensional recovery.

Patients in the CVA group showed significant improvements in local functional recovery, particularly arousal (from 0.63 to 0.80, Δ = 0.17) and motor (from 0.35 to 0.55, Δ = 0.20) functions (*p* < 0.01). However, verbal communication function showed minimal change (from 0.05 to 0.15, Δ = 0.10, *p* = 0.12), indicating persistent language dysfunction. The postoperative radar chart exhibited significant expansion along the motor and arousal axes, whereas the language communication axis demonstrated only slight alterations.

In the AIE group, recovery progression was limited; aside from arousal function (0.48–0.61, Δ = 0.13, *p* = 0.08), the change in other sub-items was <0.05 (e.g., motor function: 0.26–0.29), indicating slow neurological rehabilitation. The high overlap of radar charts, with a substantial overlap rate of preoperative and postoperative radar areas, suggested stagnant overall recovery (Table 4).

**Table 4:** Mixed-effects model analysis: changes in entropy values under anesthesia across different etiology groups.

Variable	Estimate	Standard error	*t*-value	*p*-value	95% Confidence Interval
Fixed effects					
Group (TBI/CVA vs. AIE)	−0.21	0.08	−2.63	0.009	(−0.37, −0.05)
Anesthesia State (Anesthesia vs. Baseline)	−0.15	0.07	−2.14	0.033	(−0.29, −0.01)
Group × Anesthesia State	0.18	0.09	2.00	0.046	(0.01, 0.35)
Random effects					
Random Intercept (Subject)	0.25	0.06	–	–	–
Residual	0.12	–	–	–	–

Group comparisons revealed that the TBI group exhibited the most substantial overall improvement (mean Δ = 0.21), significantly surpassing those of the CVA (Δ = 0.15) and AIE (Δ = 0.03) (*F*[2,36] = 9.84, *p* < 0.001, one-way ANOVA) groups. The post-hoc Tukey test further confirmed significant differences between the TBI and CVA groups (*p* = 0.02), as well as the AIE group (*p* < 0.001).

### Heterogeneity analysis by etiology

The pronounced differences observed among the TBI, CVA, and AIE subgroups in terms of neurophysiological responses (Figures 2–5; Table 2) and clinical outcomes (Figures 6A,B; Tables 2, 3) highlight the significant etiological heterogeneity present within the DoC cohort. Statistical analyses consistently revealed that both the extent and pattern of propofol-induced EEG modulation, such as prefrontal-parietal β/γ suppression in TBI/CVA compared to reduced frontal α in AIE, along with ΔPE differences. And subsequent functional recovery, as measured by CRS-R total and sub-score improvements, were dependent on the underlying etiology. This analysis of heterogeneity confirms that the distinct pathophysiological mechanisms associated with TBI, CVA, and AIE play a critical role in shaping both the brain’s response to anesthetic challenges and its potential for recovery following neuromodulation.

### The relationship between PE and CRS-R scores in the α band

**Figure 7:**
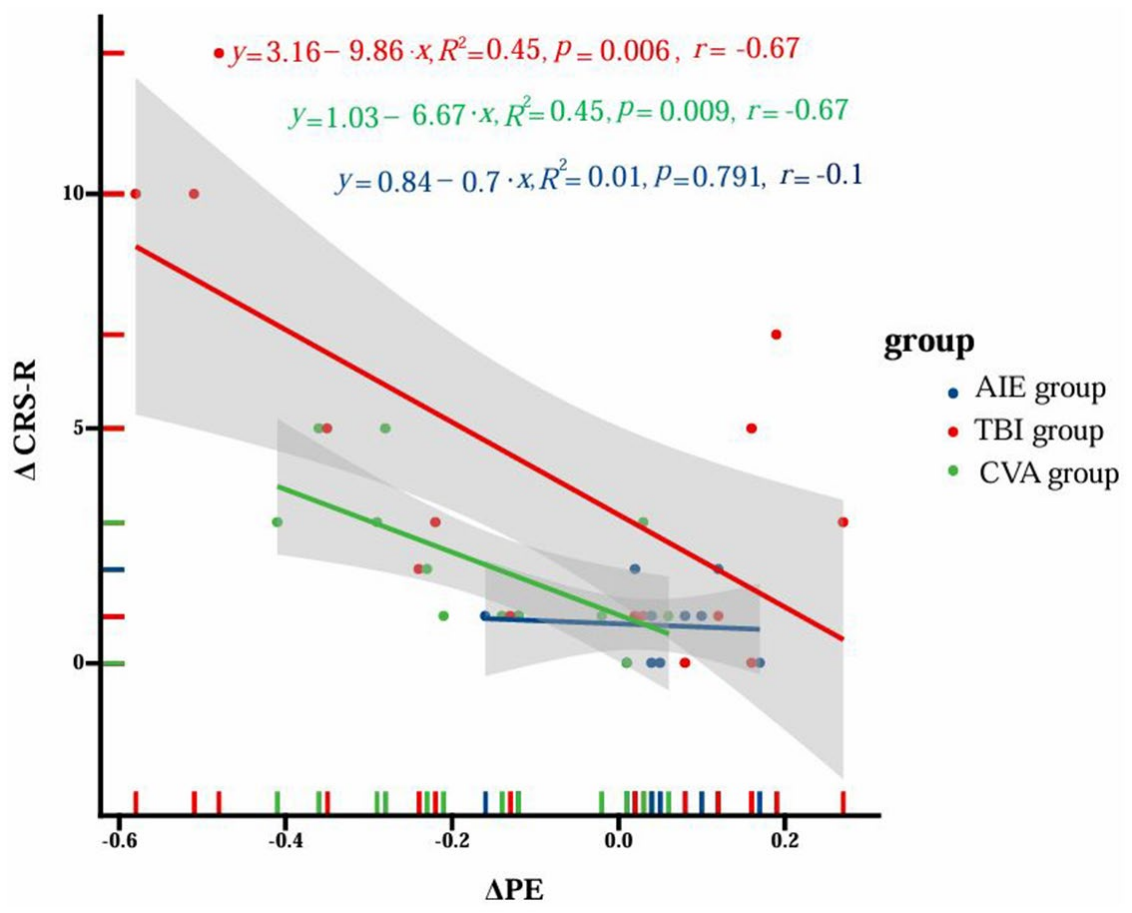
Correlation between ΔPE of α band and ΔCRS-R scores in different groups. The figure illustrates the correlation analysis between the change in permutation entropy (ΔPE) and the change in Coma Recovery Scale-Revised (ΔCRS-R) scores among three distinct patient groups with disorders of consciousness (DoC): anoxic-ischemic encephalopathy (AIE), traumatic brain injury (TBI), and cerebrovascular accident (CVA). The x-axis represents ΔPE values, while the y-axis corresponds to ΔCRS-R scores. Data points for each group are distinguished by color (blue for AIE, red for TBI, and green for CVA). For each group, trend lines are depicted alongside their respective equations, *R*^2^ values, *p*-values, and correlation coefficients (*r*). The TBI group demonstrates a significant negative correlation (*y* = 3.16–9.86x, *R*^2^ = 0.45, *p* = 0.006, *r* = −0.67), as does the CVA group (*y* = 1.03–6.67x, *R*^2^ = 0.45, *p* = 0.009, *r* = −0.67). Conversely, the AIE group shows no significant correlation (*y* = 0.84–0.7x, *R*^2^ = 0.01, *p* = 0.791, *r* = −0.1). The shaded regions surrounding the trend lines denote the 95% confidence intervals. This visualization underscores the distinct correlation patterns between ΔPE and ΔCRS-R scores across different etiological subgroups of DoC patients.

In the TBI group, there was a significant negative correlation between ΔPE in the α band and ΔCRS-R scores (*y* = 3.16–9.86x, *R*^2^ = 0.45, *p* = 0.006, *r* = −0.67). The CVA group showed a similar negative correlation (*y* = 1.03–6.67x, *R*^2^ = 0.45, *p* = 0.009, *r* = −0.67). However, no significant correlation was found in the AIE group (*y* = 0.84–0.7x, *R*^2^ = 0.01, *p* = 0.791, *r* = −0.1). Figure 7 illustrates these correlation results.

### The findings of the mixed choice model analysis

The mixed choice model analysis revealed significant differences in entropy change under anesthesia between etiological groups (TBI/CVA vs. AIE). The TBI/CVA group showed a 0.21 lower entropy change than the AIE group (estimate = −0.21, SE = 0.08, *t* = −2.63, *p* = 0.009, 95% CI −0.37 to −0.05), indicating a more pronounced reduction in entropy under anesthesia. Overall, entropy was significantly lower in the anesthetized state compared to baseline, with a mean change of −0.15 (estimate = −0.15, SE = 0.07, *t* = −2.14, *p* = 0.033, 95% CI −0.29 to −0.01). The interaction term analysis showed a significant difference in entropy change under anesthesia between the TBI/CVA and AIE groups, with an interaction estimate of 0.18 (SE = 0.09, *t* = 2.00, *p* = 0.046, 95% CI 0.01–0.35). This finding highlights the varied responses to anesthesia across different causes. The model fit well, showing some individual variability with a random intercept standard error of 0.25 and residuals standard error of 0.12.

## Discussion

In this study, we used 19-lead EEG monitoring to comprehensively investigate alterations in EEG activity before and after the administration of low-dose propofol anesthesia in patients with TBI, AIE, or CVA. The high resolution of 19-lead EEG monitoring makes it an ideal method for capturing detailed information on brain electrical activity. This approach is particularly valuable for assessing the level of consciousness in patients with DoC (39–43).

Whole-brain topography analysis provides a macroscopic view of the changes in EEG activity in patients with TBI, AIE, and CVA. The results of this study demonstrated significant decreases in the power spectra of β- and γ-waves following low-dose propofol anesthesia. These waves are closely associated with cognitive function, attention, and levels of consciousness (44, 45). Decreased β-wave power could reflect a slowing of cognitive processing speed, while reduced γ-wave power may indicate disruptions in higher neurological functions, such as working memory and information integration. Specifically, a reduction in power around 30 Hz suggests slowed cognitive processing, whereas the decline in γ-wave power (30–100 Hz) highlights potential impairments in more complex cognitive functions (46). Our integrated analysis of PSD and PE underscores the limitations inherent in traditional spectral methods. While PSD alone suggested a global suppression of β/γ oscillations across all groups, PE revealed etiology-specific variations in neurodynamic complexity. Specifically, patients with AIE demonstrated a minimal reduction in entropy (ΔPE = −0.05), indicative of rigid and low-adaptability neural networks, a nuance not captured by PSD alone. Conversely, patients with TBI or CVA showed significant declines in prefrontal entropy (ΔPE = −0.21), which were correlated with CRS-R improvements. Therefore, our integration of PSD and PE supports the recent advocacy for multidimensional biomarkers in determining the prognosis of DoC (47, 48). Specifically, frontal β/γ reactivity, as measured by PSD, may indicate residual network integrity, while PE assesses functional flexibility. This dual signature is critical for predicting responsiveness to neuromodulation interventions. In contrast, minimal variations were observed in the power of θ- (4–7 Hz), δ- (1–3 Hz), and α-waves (8–12 Hz) before and after anesthesia, suggesting the lower correlation of these frequency bands with the effects of low-dose anesthesia.

Anesthetic agents alter EEG frequency bands that correspond to different states of consciousness. θ-waves are typically associated with deep sleep and certain cognitive processes, while δ-waves are linked to slow-wave sleep or pathological conditions following brain injury (49). In contrast, α-waves are generally associated with relaxation and closed-eye brain states (50). The stability in these frequency bands may indicate that fundamental brain rhythms and intrinsic functional patterns retain a degree of stability, even under the influence of anesthetics, while neural activity in specific brain regions is more significantly affected. The marked reductions in β- and γ-wave power observed in patients with TBI, AIE, and CVA could be attributed to the direct effects of anesthetics on the cerebral cortex and subcortical networks. Propofol, a GABA receptor agonist, may contribute to decreased high-frequency EEG activity by enhancing inhibitory neurotransmission within the central nervous system (50). This reduction may also be associated with anesthetic-induced changes in neural network connectivity, which affect the brain’s capacity to transmit and integrate information across different regions and networks (51). The differential alterations in PE observed across various etiological subgroups provide further insights into the pathophysiological mechanisms underlying DoC. The strong negative correlation between ΔPE and ΔCRS-R in the TBI/CVA group (*r* = −0.67, *p* < 0.01) underscores the clinical importance of reduced entropy, indicating that propofol may temporarily suppress pathological oscillations and support neuroplasticity. Conversely, the absence of this correlation in the AIE group (*r* = −0.1, *p* = 0.791) aligns with its irreversible damage, emphasizing the prognostic value of entropy dynamics. In the TBI and CVA groups, the marked reduction in prefrontal entropy (ΔPE = −0.21) indicates propofol-induced suppression of neural complexity, which was paradoxically correlated with enhanced CRS-R scores. The causal relationship between PE reduction and clinical improvement is uncertain. This may result from propofol directly inhibiting pathological neural oscillations or from SCS activating networks affected by propofol-induced neurosuppression. This phenomenon may suggest that anesthesia temporarily mitigates maladaptive hyperconnectivity or chaotic neural activity, thereby enabling residual neural networks to re-establish functional hierarchies. The decrease in entropy values following anesthesia may reflect a shift from disorganized, high-variability neural states to more ordered dynamics, thus facilitating thalamocortical integration essential for consciousness recovery. In contrast, the minimal change in entropy observed in the AIE group (ΔPE = −0.05) highlights the severe disruption of neural dynamics resulting from global hypoxic injury, which limits the potential for functional reorganization. The maintenance of entropy levels in the AIE group is consistent with their stagnant recovery, as rigid, low-complexity networks lack the adaptability necessary for neuromodulation-driven plasticity. The etiology-specific entropy dynamics underscore the utility of PE as a sensitive indicator of neural adaptability and recovery potential. This information complements spectral analyses by encapsulating the nonlinear temporal characteristics of brain activity.

The administration of anesthetic drugs imposes an additional burden on the residual brain activity of patients with impaired consciousness. Therefore, assessing the brain’s response to these drugs can offer valuable insights into cognitive function and levels of consciousness. Propofol, a commonly used intravenous anesthetic in clinical practice, may not cause sedation at low doses in healthy individuals but instead can cause behavioral arousal and activation, characterized by an EEG pattern of increased β-wave (12.5–25 Hz) activity and decreased slow-wave activity (52, 53). In contrast, anesthetic drugs place stress on the brain in patients with impaired consciousness, and the ability to endure this stress can serve as a functional test for to assess the potential for consciousness recovery (54). A large multicenter trial by Ruijter et al. demonstrated that propofol did not affect the prognostic value of early EEG despite influencing the amplitude, background continuity, and dominant frequency of early EEG readings (55).

Various anesthetic agents alter EEG activity differently. Anesthetics like propofol and sevoflurane boost α oscillations (8–12 Hz) in the frontal region during unconsciousness induction (56, 57), indicating sufficient anesthetic depth and reduced postoperative cognitive risks (58). α-band activity correlates with anesthesia depth indices but can decrease with surgical stimulation (57, 58), suggesting cortical arousal (59). In the *δ* frequency band (0.5–4 Hz), power generally increases during anesthesia maintenance, especially in the frontal region (60). Sevoflurane significantly raises δ-band power, while propofol enhances δ waves globally and extends microstate durations (61). Propofol anesthesia involves δ-α cross-frequency coupling, where δ wave phases affect α wave amplitudes, likely contributing to unconsciousness (62). Sevoflurane and propofol both increase frontal *θ*-band power (60, 63), with θ-band activity sensitive to sensory input. Frequency ratios like δ/α and (δ + θ)/(α + β) help measure anesthesia depth and brain function, warning of conditions like brain herniation (64). EEG patterns differ among anesthetics and are complicated by drug combinations. Frontal α oscillations indicate anesthesia depth (56, 57), while δ activity links to unconsciousness (61). The variability in frequency modulation patterns across different anesthetics indicates the necessity for personalized EEG data analysis to enhance the accuracy of anesthesia depth monitoring.

The observed variations in CRS-R outcomes 3 months postoperatively correspond with etiology-specific EEG reactivity patterns under propofol anesthesia. The TBI cohort exhibited the most significant recovery, with four patients progressing to EMCS. This outcome is likely due to the preserved neuroplasticity associated with trauma-induced DoC (18, 19). In TBI, diffuse axonal injury tends to spare subcortical arousal networks, thereby facilitating functional reorganization through SCS-mediated neuromodulation. This is further supported by the results of the radar chart analysis in the present study, which indicated multidimensional recovery across the motor, visual, and auditory domains, suggesting the reactivation of thalamocortical and frontoparietal circuits essential for integrative consciousness. In contrast, the CVA group demonstrated specific improvements in arousal and motor function but limited progress in communication abilities, indicative of lesion-specific network constraints (65). Ischemic and haemorrhagic injury disrupt localized cortical–subcortical pathways, resulting in residual networks that are only partially responsive to neuromodulation. The attenuation of prefrontal β/γ activity observed following propofol administration may suggest the preservation of frontal executive circuitry, which facilitates partial recovery (44, 45). However, persistent language deficits underscore the susceptibility of the perisylvian regions to vascular injury, thereby constraining comprehensive recovery. Conversely, the stagnation in consciousness and CRS-R sub-score recovery observed in the AIE group in the present study highlight the devastating impact of global hypoxic-ischaemic damage (66, 67). Extensive cortical laminar necrosis and thalamic degeneration inhibit the neurodynamic flexibility required for propofol-induced network reorganization (68–72). The negligible reduction in PE (−0.05) and the stability of θ/δ power post-anesthesia further indicate a rigid, low-complexity brain state that is resistant to neuromodulatory interventions. Importantly, the observed correlation between reduced PE (ΔPE = −0.21) and improvements in CRS-R scores among patients with TBI or CVA indicates that anesthesia-induced neurosuppression may ‘unmask’ latent functional networks, thereby preparing them for plasticity driven by SCS. This finding is consistent with those of previous studies associating prefrontal β/γ reactivity with cognitive reintegration in DoC (73–75). In contrast, the absence of these dynamics in patients with AIE suggests irreversible network fragmentation, highlighting the need for etiology-specific prognostic frameworks. The robust negative correlation between reduced PE and improved CRS-R (*r* = −0.67) suggests that patients with higher baseline neural complexity, such as those with TBI or CVA, derive greater benefits from neuromodulation. This effect is potentially mediated through anesthesia-facilitated stabilization of neural networks. This observation is consistent with the “network reset” hypothesis, which posits that transient neurosuppression may enhance brain plasticity by mitigating pathological oscillations. The significant decline in PE observed in patients with TBI or CVA may indicate the selective inhibition of dysfunctional circuits, thereby revealing latent functional connections that facilitate recovery. Conversely, the static entropy profile observed in patients with AIE suggests irreversible network fragmentation, where neither suppression nor activation can induce meaningful reorganization. These findings underscore that anesthesia-induced modulation of entropy is not merely a passive biomarker but may actively influence post-SCS neuroplasticity in ways that are dependent on the underlying etiology.

This study identified significant heterogeneity in neurophysiological responses induced by propofol and subsequent recovery following SCS among patients with varying etiologies, including TBI, CVA and AIE, through stratified analyses. This heterogeneity is attributed to etiology-specific pathological mechanisms: patients with TBI or CVA exhibit partial thalamocortical network plasticity, with a notable reduction in prefrontal entropy (ΔPE = −0.21) significantly correlating with improvements in the CRS-R scores (*p* = 0.003). This suggests that propofol may temporarily “reset” pathological network oscillations, thereby enhancing the efficacy of SCS. In contrast, patients with AIE, who experience global hypoxic–ischemic injury, demonstrate rigid neurodynamics (ΔPE = −0.05, *p* = 0.12), with no observed correlation between changes in entropy and clinical recovery. These findings highlight the importance of etiological stratification in prognostic evaluations. By integrating multimodal EEG metrics, such as PSD and PE, the vulnerability of neural networks can be quantified, providing a theoretical foundation for precision neuromodulation, particularly for prioritizing TBI and CVA patients. Future multicenter studies are necessary to validate heterogeneity thresholds and to develop stratified clinical treatment strategies.

This single-center retrospective study is subject to several limitations. The relatively small sample size (*n* = 40) may constrain the statistical power and limit the generalizability of the findings. The study did not address etiological heterogeneity within the CVA cohort, as ischemic and hemorrhagic subtypes were not analyzed separately, which may obscure pathology-specific neural responses. The follow-up period of 3 months may be inadequate to differentiate between therapeutic effects and natural recovery dynamics. Importantly, the concurrent administration of SCS and rehabilitation therapy prevents the isolation of SCS-specific treatment effects due to the lack of a rehabilitation-only control group. Additionally, the study did not quantify the dynamic interaction between the neuroinhibitory effects of propofol and SCS-mediated neuromodulation, nor did it include a propofol-only control group—an omission driven by ethical considerations but one that limits mechanistic interpretation. Lastly, although independent component analysis was employed to mitigate artifacts, the EEG findings lack validation through multimodal neuroimaging techniques (e.g., rs-fMRI or diffusion tensor imaging), which could substantiate the functional or structural correlates of entropy changes. The lack of simultaneous fMRI or DTI data prevents us from confirming if PE changes are due to structural connectivity alterations or functional network reorganization, thus limiting the understanding of the link between entropy reduction and clinical outcomes. In future studies, we will extend patient follow-up to 12 months to better evaluate long-term neuroplasticity and recovery. Additionally, we’ll refine the study design by using multivariate regression models to control for confounding factors like age, disease duration, and CRS-R score, enhancing result accuracy and comparability.

## Conclusion

This study analyzed EEG responses following the administration of low-dose propofol to patients with DoC stemming from various aetiologies. The results demonstrated significant reductions in whole-brain β and γ wave power, whereas θ-, δ-, and α-wave activities remained relatively stable. Notably, patients with AIE exhibited decreased frontal α-wave power, whereas those in the TBI and CVA groups showed pronounced alterations in α, β, and γ waves within specific brain regions. Furthermore, some patients within the TBI and CVA cohorts experienced improvements in consciousness 3 months post-surgery. These findings suggest that EEG response patterns may reflect the integrity of residual brain networks and the potential for recovery. Additionally, frontal β/γ reactivity might serve as a potential indicator for neuromodulation therapy in patients with DoC due to specific aetiologies. Significantly, the observed reduction in EEG complexity (entropy) induced by propofol was associated with the context of SCS therapy. However, the design of this study does not establish a direct causal relationship between the propofol-induced changes in entropy and the enhanced efficacy of SCS. Further validation is required to determine its predictive accuracy and applicability in clinical practice. Future studies with well-designed control groups are needed to clarify the interaction between propofol, SCS, and rehabilitation therapy on consciousness recovery.

## Data Availability

The original contributions presented in the study are included in the article/supplementary material, further inquiries can be directed to the corresponding author.

## Footnotes

^1^ https://www.r-project.org
